# Remote group intervention for adults with cancer-related cognitive impairment: a feasibility study

**DOI:** 10.1007/s00520-025-10114-7

**Published:** 2025-12-04

**Authors:** Chenanit Hamami, Tamar Peretz, Mor Nahum, Talia Maeir, Ofra Maimon, Yafit Gilboa

**Affiliations:** 1 School of Occupational Therapy, Faculty of Medicine, Hebrew University of Jerusalem, 91240 Jerusalem, Israel; 2 Department of Physical Medicine and Rehabilitation, Hadassah Medical Center, POB 24035, 91240 Jerusalem, Israel; 3 Hadassah Medical Center, Jerusalem, Israel; 4 Faculty of Medicine, Hebrew University, Jerusalem, Israel; 5 Departments of Psychology and of Cognitive Science, The Hebrew University of Jerusalem, Mt. Scopus, 91905 Jerusalem, Israel; 6 Sharett Institute of Oncology, Hadassah-Hebrew University Medical Center, 91120 Jerusalem, Israel

**Keywords:** Occupational therapy, Tele-rehabilitation, Chemobrain, Computerized cognitive training, Group therapy, Occupation-based intervention

## Abstract

**Purpose:**

This study aimed to examine the feasibility of a remote group intervention, Computerized Retraining and Functional Treatment Group (CRAFT-G), among adults with cancer-related cognitive impairment (CRCI). CRAFT-G is designed to improve occupational performance, objective and subjective cognitive functions, quality of life (QoL), and sense of loneliness.

**Method:**

A single-arm quasi-experimental design using mixed quantitative and qualitative methods was applied. Five cancer survivors with CRCI, aged 30 to 57 years, received the CRAFT-G intervention, which includes 6 weekly group sessions along with 18 sessions of individualized computerized cognitive training (CCT; 3 per week). Feasibility was measured in terms of recruitment, retention, adherence, acceptability, and potential effectiveness. Outcome measures were administered at baseline and post intervention.

**Results:**

The recruitment rate was 35% (6/17), and the retention rate was 83.2%. Partial adherence was found to the group sessions (80%) and to the CCT (40%). Participants expressed moderate to very high satisfaction with the intervention. Three main themes emerged from the feedback interviews: (a) group dynamic and therapeutic context, (b) intervention components, and (c) client factors. Significant improvements in occupational performance and clinically significant improvements in self-perceived cognitive function were also found, demonstrating potential effectiveness.

**Conclusion:**

The results support the feasibility and potential effectiveness of the CRAFT-G among adults with CRCI. A controlled, larger trial should be conducted to validate these results.

**Implications for cancer survivors:**

A combined intervention approach of an occupation-based intervention along with cognitive training and psychoeducation about CRCI applied in remote group format may be considered when treating adults with CRCI.

**Supplementary Information:**

The online version contains supplementary material available at 10.1007/s00520-025-10114-7.

## Introduction

Cancer survival rates have increased dramatically in recent years, causing a conceptual shift from an acute illness to a chronic health condition [1]. Up to 80% of cancer survivors report cognitive deficits as a result of the disease and its treatment, known as cancer-related cognitive impairment (CRCI) [2]. CRCI is defined as mild to moderate cognitive deficit in cancer survivors with non-central nervous system (non-CNS) malignancies. It affects working memory, attention, executive functions, language, and speed of processing [3, 4]. Long-term survivors may face social, economic, psychological, employment, and health-care issues, in addition to secondary effects from the treatments [5]. To date, there are limited evidence-based treatments and services suggested for adults with CRCI [6–11].

Computerized cognitive training (CCT) aims at improving, maintaining, or restoring isolated cognitive functions, based on principles of perceptual learning and neuroplasticity; this is achieved by repeatedly practicing graded tasks adapted to an individual’s performance [12, 13]. Several studies have shown that CCT among cancer survivors with CRCI improves cognitive functions such as attention and memory [14–17], verbal memory learning [18, 19], speed of processing [16, 20], and executive functions [14, 20]. Furthermore, studies found CCT-related improvements in survivors’ self-perceived cognitive functioning [14, 21, 22], in quality of life (QoL) [16], as well as reduced depression, anxiety, and fatigue [20, 21].

Currently, CCT is showing great promise as an effective intervention approach being studied for CRCI and shows sufficient evidence in positive cognitive and emotional outcomes to suggest clinical application [23, 24]. However, patient adherence to CCT is low [25, 26]; and when applied in isolation, CCT usually neither does not lead to improvements in broader cognitive tasks beyond those directly trained nor does it enhance everyday performance or increase QoL [27, 28]. Therefore, a systematic review regarding evidence-based cognitive rehabilitation recommended integrating CCT with individualized functional interventions that focus directly on treating daily challenges [29]. Moreover, a review among breast cancer survivors suggested that combining cognitive and compensatory strategy training may have beneficial effects for improving everyday functioning and QoL [9].

Cognitive Orientation to Occupational Performance (CO-OP) [30] is a client-centered approach aimed at improving performance in daily function by using meta-cognitive strategy acquisition, in order to foster generalization and strategy transfer to different occupational contexts. In general, CO-OP utilizes a main executive global strategy: “Goal-Plan-Do-Check” (GPDC), for problem-solving in an occupational challenge chosen by the client [31]. The framework was originally developed for children with developmental coordination disorder, and it has been adapted for use in various populations across ages [30, 32, 33]. Maeir and colleagues [26] developed a telerehabilitation protocol for adults with CRCI: the Computerized Retraining and Functional Treatment (CRAFT). The program provides individualized treatment and combines CCT intervention with the CO-OP approach. The results of their study showed the feasibility of the intervention and its potential effectiveness to enhance participation in daily life, improve objective cognitive performance, and reduce subjective cognitive impairment. However, no effect was found on QoL [26, 34].

The chronic challenge facing cancer survivors with CRCI highlights the growing need for long-term rehabilitation options [35]. Moreover, many of the cancer survivors return to participate in their previous occupations, so there is added value in finding a treatment solution that is accessible and flexible for them [36, 37]. Telerehabilitation provides a pragmatic solution for reducing health-care costs, treatment time, and hospitals visits [38, 39]. Moreover, it is an accessible and effective solution for cancer survivors with CRCI that allows them to maintain their daily routine [36].

Feelings of loneliness and societal stigma are described as having a negative impact on the daily function and QoL of cancer survivors [40]. Moreover, loneliness has been identified as a potential risk factor for the appearance of CRCI symptoms [41]. Therefore, group-based interventions for cancer survivors were shown to provide an abundance of therapeutic benefits, including opportunities for social support and interpersonal learning, along with instilling new hope by normalizing their experience [35, 42]. Indeed, cognitive intervention among cancer survivors with CRCI in a group setting may be more appropriate than individual intervention, since it may simultaneously promote the two treatment goals of cognitive rehabilitation and reduced feelings of loneliness [43].

The CRAFT Group (CRAFT-G) intervention incorporates four components: the CCT [23, 24], the CO-OP functional intervention [26, 34, 44], the telerehabilitation format [26, 34], and the group therapy [42, 45, 46]; all of which have been demonstrated to be effective and are recommended for CRCI.

The aim of the current study was to determine the feasibility of the CRAFT-G intervention and provide preliminary data regarding its potential efficacy in preparation for a large-scale trial. The specific objectives of the study were as follows: (1) to examine recruitment, retention, adherence, and satisfaction of the participants with the program; (2) to evaluate the potential effect of the CRAFT-G on the participants’ occupational performance, cognitive function, QoL, and sense of loneliness.

We hypothesized that (1) the CRAFT-G will be found feasible, and participants will be highly satisfied; (2) the outcome measures will show clinically and statistically significant improvements in occupational performance, cognitive function, QoL, and personal sense of loneliness. The CRAFT-G intervention proposed in this study has the potential for creating a cost-effective, generalizable treatment that bridges current therapeutic gaps for CRCI, which currently impairs the lives of millions worldwide.

## Methods

### Study design and participants

A single-arm quasi-experimental design using mixed quantitative and qualitative methods was applied.

Participants (*N* = 5) were adult cancer survivors. All participants were recruited using a convenience sample via social media networks. Inclusion criteria were as follows: (a) aged 18 to 65 years; (b) subjective concerns about decline in cognitive functioning related to the diagnosis of cancer and/or cancer-related treatment, determined by an affirmative answer to the question: “Do you have concerns about your memory or other thinking abilities following cancer treatment?” [26]; (c) completion of active treatment for non-CNS cancer (e.g., surgery, chemotherapy, radiation, hormonal therapy, and/or biological treatment) at least 6 months prior to recruitment; (d) daily access to a computer and Internet connection; (e) able to sign an informed consent; (f) no known psychiatric background (according to self-report); (g) fluent in Hebrew; (h) no major cognitive decline (Montreal Cognitive Assessment; [MOCA] > 19; [47]).

#### Sample size considerations

Sample size considerations were based on previous feasibility studies that initially examined interventions based on the CO-OP approach applied a similar set-up, which included a sample of five to six participants [26, 32].

### Instruments

#### Descriptive and demographic measures

*A demographic and medical questionnaire* was developed and designed for the study to collect descriptive personal, demographic, and medical information. Filling out the questionnaire took about 5 min.

#### Feasibility measures

##### Recruitment, retention, and adherence

*Therapist logs* recorded information regarding the number of eligible participants, recruitment procedure, and retention.

##### Acceptability

Post intervention, the participants filled out a *questionnaire* aimed at assessing their satisfaction with the CRAFT-G. The questionnaire was designed for the study of Maeir and colleagues [26] and was adapted to group intervention; it consists of 15 items on a 5-point scale (1 = “to a very little extent” to 5 = “to a very large extent”). In addition, semi-structured *feedback interviews* of 20–60 min were conducted post intervention to obtain the participants’ perceptions of the CRAFT-G intervention. The interviews were performed by an assessor who was not involved in the intervention; they were audio-recorded and later transcribed.

#### Potential effectiveness measures

Validated Hebrew translations were used for all standard questionnaires.

##### Primary outcome

These measures assessed activity performance in the participants’ chosen goals.

*The Canadian Occupational Performance Measure* (COPM) [48] is a semi-structured interview that examines changes in occupational performance and satisfaction with performance in daily functioning, by rating the daily activities that are most important to the participant. Following identification of functional difficulties (potential goals) in the various domains of occupation, the participants are asked to rate them by level of importance on a 10-point scale (from 1 = “low importance” to 10 = “great importance”). After identifying the three most important problems, the participant rates each problem on a 10-point scale indicating their level of performance (1 = “not able to do it at all” to 10 = “able to do it extremely well”) and satisfaction (= “not satisfied at all” to 10 = “extremely satisfied”). A change score of 2 or more points is considered a minimal clinically important difference (MCID) [49]. The COPM is used in a variety of populations, including cancer survivors with CRCI [42, 44]. The tool has shown strong test–retest reliability [50], good concurrent validity with other functional outcome measures, and excellent content and face validity [49]. The interview takes about 20 min.

*The Performance Quality Rating Scale* (PQRS) [51] is an observational tool designed to complement the assessment of the participants’ occupational performance in the goals chosen in the COPM [48]. The purpose of the PQRS is to provide an objective measure of occupational performance from the therapist’s point of view. The therapist rates the goal performance by observation if possible, or by the participant’s report, based on the COPM 10-point rating scale (1 = “not performed at all” to 10 = “performed well”) [52]. The PQRS has good inter-rater reliability, 0.71 to 0.77, and good test–retest reliability, > 0.80. However, convergent validity with the COPM is inconsistent, and further validation is required [51].

##### Secondary outcomes

These measures assessed cognitive function, quality of life, and personal sense of loneliness.

*The Adaptive Cognitive Evaluation* (ACE; https://neuroscape.ucsf.edu/researchers-ace/) is an online cognitive assessment battery for objectively assessing cognitive domains, using four tasks that test dual task, inhibition, information processing speed, mental flexibility, selective attention, and interference resolution (see Supplementary Table S1 for a description of the subtest battery used in the study). The ACE is used among different populations, with different versions for children and adults. The tool has been tested for construct validity [53] and is currently undergoing large-scale norming and validation [54]. However, to date, there are no psychometric indices available. The duration of the assessment is about 30 min.

*The Functional Assessment of Cancer Therapy—Cognition* (FACT-Cog) [55] is a self-report questionnaire that examines a participant’s subjective perception of cognitive functions. The questionnaire was developed for cancer patients with cognitive function issues and includes 37 items that assess memory, attention, language, and thinking abilities. The items are divided into four subscales: perceived cognitive impairment (PCI), perceived cognitive abilities (PCA), comments from others (OTH), and impact on quality of life (QoL). The participants are asked to rate the items on a five-level scale ranging from 0 = “never” or “not at all” to 4 = “several times a day” or “very much;” higher scores represent better perceived cognitive function and QoL. MCID for the FACT-Cog was found to range from ‏ + 4.7% to ‏ + 7.2% for improvement [56], and −6.9 to −10.6 points for reduction [57]. The tool is considered valid and is widely used among cancer patients [58, 59]. High internal reliability was found (*α* = 0.89) [60]. A correlation was found between the FACT-Cog and other measures of verbal memory and executive functions (*r* = 0.23–0.25), as well as tools identifying symptoms of depression, fatigue, and anxiety (*r* = 0.54–0.6) [59]. Completion takes 10–15 min.

*The Functional Assessment of Cancer Therapy—General Practice* (FACT-GP) [61] is a self-report questionnaire for assessing QoL; it was designed for adult cancer survivors. The questionnaire consists of 21 items on a 5-point frequency scale (0 = “not at all” to 4 = “to a very high extent”). The items are divided into four domains: physical condition, social/family condition, emotional condition, and functional status. A higher score indicates a higher level of QoL. The tool was found to be reliable, with moderate-to-high internal consistency (*α* = 0.65–0.89) and high test–retest reliability (from 0.82 to 0.92) between repeated transfers of 3–7 days. The questionnaire was found to have high convergent validity (*r* = 0.69–0.89), comparable to tools that measure functional status and QoL. In addition, the FACT-GP distinguishes between subjects with disease stages of different severity levels and subjects in recovery stages [61]. Filling out the questionnaire takes about 5 min.

*The revised UCLA Loneliness Scale* (UCLA) [62] is a commonly used self-report questionnaire for measuring loneliness. It includes 20 descriptive items that reflect the participant’s degree of satisfaction with their social relationships on a 4-point scale (1 = “never” to 4 = “often”), with a higher score indicating greater loneliness. The instrument has been used in studies conducted among cancer patients and survivors [41, 63]. The internal reliability of the Hebrew version [64] was high (*α* = 0.9) [65]. Convergent validity for the scale showed significant correlations with the NYU Loneliness Scale (*r* = 0.65) and the Differential Loneliness Scale (*r* = 0.72) [66]. Filling out the questionnaire takes about 3–5 min.

### Procedure

Ethics approval was obtained from the Hadassah Helsinki Ethics Committee (protocol no. 0124–22-HMO). Recruitment was conducted via advertisement on social media networks. Interested individuals were screened in a telephone interview, and a face-to-face meeting was scheduled with eligible individuals. Following informed consent, participants underwent baseline assessment in individual in-person meetings. Post-intervention assessments were conducted remotely, with participants completing the ACE and the questionnaires on their personal computers. The feedback interview was conducted by an occupational therapist who did not take part in the intervention. The baseline and post-intervention assessments lasted between 90–120 min and 60–100 min, respectively. The study procedure is presented in Supplementary Figure S1.

#### CRAFT-G intervention protocol

The intervention (see Supplementary Table S2) was administered remotely in accordance with the therapeutic protocol described in a previous study [26], while adapting the format of the therapeutic meeting to a group setting. In general, the intervention lasted 6 weeks, and each remote CO-OP group session lasted 90 min. Between the group sessions, the participants were asked to perform three CCT training sessions per week (approximately 30 min each). In total, the participants were asked to complete 9 h of CCT by the end of the intervention.

The intervention included a combination of four components: 

1. CCT [67]: The participants practiced CCT using an experimental application game (not commercially available) called Legends of Hoa’manu (LoH). LoH was developed by Zaffiria (https://www.zaffiria.it/legends-of-hoamanu/) as part of the Depression Stimulation Cognitive Control Video game Remotely (DiSCoVeR) project, an Era-Net Neuron funded project, supervised by Prof. Daphne Bavelier (University of Geneva) and Prof. Mor Nahum (Hebrew University of Jerusalem). The application is user-friendly, employing video game features, and met the cognitive training requirements for adults with CRCI. It is designed for the practice of cognitive skills, such as working memory, inhibition, concentration, divided attention, attentional control, mental flexibility, and executive functions. In each game, training progresses in an adaptive, individualized manner, ensuring that every participant is optimally challenged [67]. The application was installed on the participants’ smartphones or computers according to their preference. Research staff were available to troubleshoot any issues that came up during the training.

2. CO-OP [30]: In each group session, the main executive global strategy (GPDC) was presented as a guiding framework for solving functional cognitive problems. In the course of the group intervention, one of the three goals that participants selected during baseline assessments was used to practice the strategy. During the sessions, the participants practiced the formation of *plans* for the *goals* they had set in the individual baseline meetings. Between the sessions, the participants were asked to *do* (carry out) the plan and report on their progress in the following sessions. The next session included monitoring the execution of the plan and *checking* whether the plan worked. If the goal was not achieved, the participant and the group, through guided discovery, identified a new potential plan, and the process was repeated [30]. Participants were encouraged to share their personal process with the group during the sessions. Throughout all sessions, the therapist encouraged generalization of skills and strategies to the natural environment, and their transfer to novel skills [68].

3. Psychoeducational content: Six short units were presented to participants in each session about common symptoms experienced by cancer survivors after recovery, and their connection to cognitive decline.

4. Group intervention: The group process was defined according to Tuckman’s stages of group formation [69]. The structure of each session was based on Cole’s OT Group framework (Seven-Step Group Process) [70], including Introduction, Activity, Sharing, Processing, Generalization, Application, and Summary.

All sessions were videotaped. Two occupational therapists (Y. Gilboa, T. Maeir), who are certified in the CO-OP approach and are experienced with the experimental approach, trained the therapist (C. Hamami) and monitored all sessions to ensure treatment fidelity.

### Data analysis

Data analysis was performed with IBM SPSS Statistics for Windows [71]. First, descriptive statistics were used to describe the characteristics of the sample, the feasibility of the study, and the outcome measures. The recruitment rate was determined by dividing the number of participants who started the intervention by the number of participants who were assessed for eligibility. The recruitment rate was considered feasible if it exceeded 15%. The retention rate was defined as the number of participants who remained in the study until the last wave of data collection, as a proportion of the total number of participants recruited at the baseline assessment. The retention rate was considered feasible if it exceeded 75% [72].

Adherence was measured separately in the assessment procedures for the CO-OP and for the CCT. Good adherence was considered to be participation in five (80%) of the CO-OP sessions and completion of all questionnaires pre and post intervention [73]. Completing 8 or more hours of CCT was considered good adherence [26], which was confirmed using data from the application’s log-in portal.

Qualitative content analysis of the feedback interviews was used to examine acceptability. Due to the small sample size, analysis of changes in outcome measures pre-post intervention was conducted by a nonparametric Wilcoxon signed-rank test. The significance level was set at 5%.

## Results

### Sociodemographic and clinical characteristics

Sociodemographic and clinical characteristics of the sample are presented in Table 1. The sample included five participants (four women), whose ages ranged from 30 to 57 years (average 46.2 ± 13.08). The cancer type varied across participants, as well as the time from active cancer treatment completion (7–132 months).

**Table 1:** Participant characteristics, adherence and acceptability (*N* = 5)

Participant		1	2	3	4	5
Personal characteristics	Sex	Female	Female	Female	Female	Male
	Age	54	30	57	56	34
	Level of education	Master’s degree	High School	Master’s degree	Doctor of Medicine	Bachelor’s degree
	Work status	Beyond Full time	Not working	Part time	Beyond full time	Not working
Medical data	Disease site	Breast	Thyroid gland and abdominal	Breast	Breast	Lymphoma Hodgkin’s
	Time since last treatment (months)	132	77	14	18	7
	Type of treatments	Radiation hormonal therapy	Chemotherapy surgery biological treatment	Surgery radiation	Chemotherapy surgery radiation hormonal	Chemotherapy
Cognitive status	MOCA score	23	27	30	30	26
Adherence	CO-OP (sessions)	6*	6*	6*	5*	3
	CCT (hours)	6:41	3:07	13:48**	09:44**	1:05
Acceptability	Overall satisfaction	4.8	4.7	4.2	2.9	3

The asterisk “*” indicated full adherence to CO-OP in five or more sessions. The asterisks “**” indicated full adherence to CCT ≥ 8 h. Satisfaction questionnaire score range: 1–5 points

### Feasibility

#### Recruitment, retention, and adherence

**Fig. 1:**
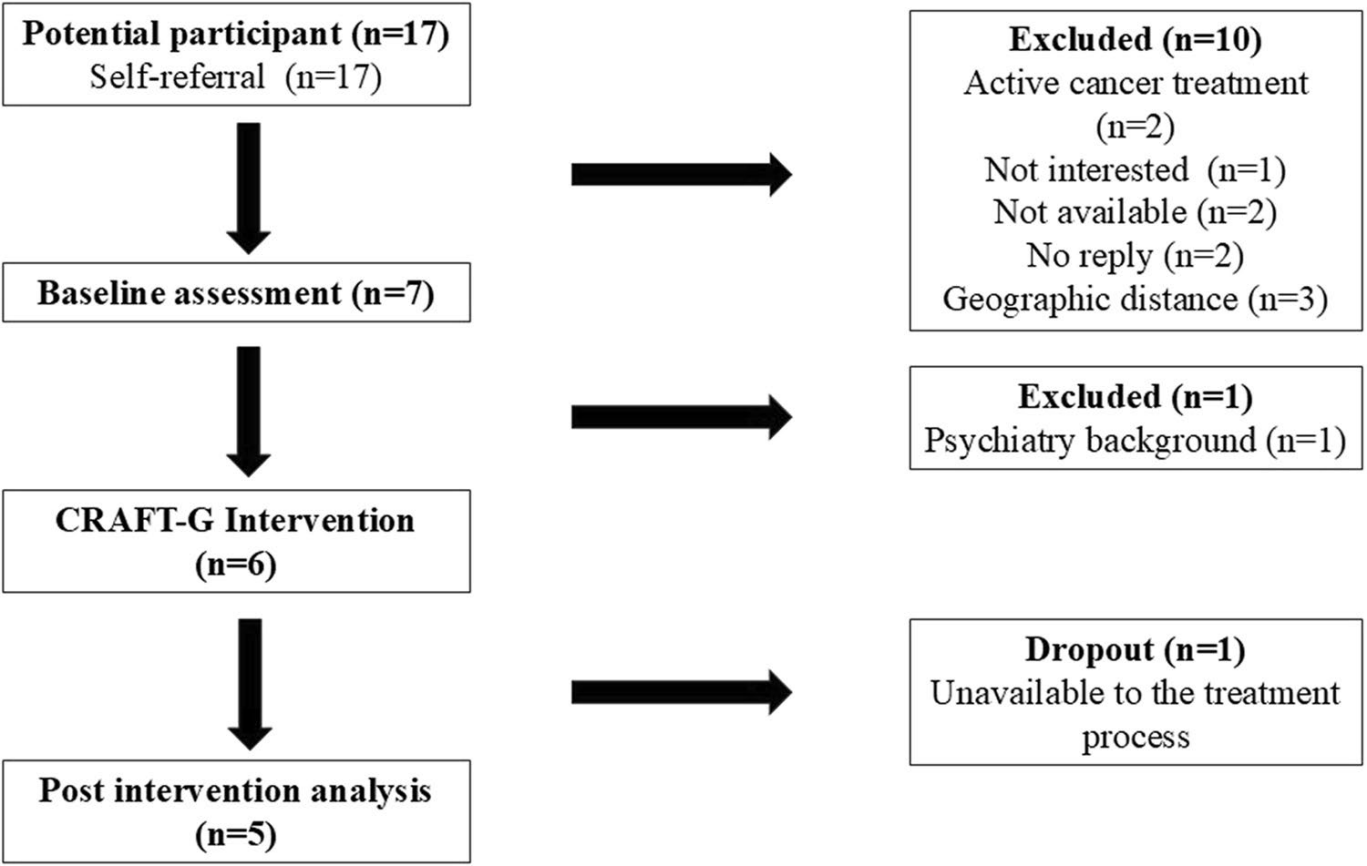
Flow diagram of the study recruitment, baseline assessements, intervention, and post-intervention analysis

Participant recruitment took place between January 2023 and April 2023. Figure 1 shows a flow diagram of the study. Seventeen potential participants showed interest in the intervention following a week of advertisement. The first seven who met the eligibility criteria and who were available for the group sessions were invited to participate in the study. One participant was excluded during baseline assessment for not meeting the study criteria. Thus, the recruitment rate was 35% (6/17). From the six participants who began the intervention, one dropped out after 2 weeks; therefore, the retention rate was 83.2%.

All outcome measures were collected according to schedule, indicating full adherence to the assessment procedure. Partial adherence was found to the CO–OP part of CRAFT-G, with four participants (80%) completing at least five sessions, and likewise for adherence to the CCT, with two out of the five participants completing the minimum required training of 8 h (see Table 1).

**Table 2:** Changes in primary and secondary outcome measures from baseline to post intervention

Outcome measure (possible range of scores)	Baseline	Post intervention			
	Median (IQR)	Median (IQR)	z	*p*	r(ES)
**COPM (occupational performance) (1–10)**					
Performance	3.30 (2.30–4)	4.70 (3.85–7.15)	2.02	**0.04**	**0.90**
Satisfaction	2.70 (1.80–3.80)	4.30 (3.20–7.65)	2.02	**0.04**	**0.90**
**PQRS (occupational performance) (1–10)**	4 (3–4.85)	5 (4–7.30)	2.03	**0.04**	**0.91**
**ACE (cognitive assessment evaluation)**					
Triangle trace (dual tasking)	622.80 (538.22–687.42)	639.05 (422.70–665.90)	−1.07	0.29	−0.48
Stroop/color tricker (response inhibition)	0.93 (0.57–1.16)	1.18 (0.54–1.85)	0.73	0.47	0.33
Sun and moon (task switching)	1.15 (0.93–1.58)	1.44 (0.95–2.02)	1.46	0.14	**0.65**
Flanker (selective attention and interference resolution)	1.57 (1.50–2.10)	1.73 (1.19–2.33)	0.37	0.72	0.16
**FACT-Cog (perceived cognitive function)**					
Perceived cognitive impairments (PCI) (0–72)	37.00 (28.50–50.50)	41.00 (29–64.50)	1.21	0.23	**0.54**
Impact of perceived cognitive impairments of quality of life (QoL) (0–16)	7.00 (3–11)	8.00 (5–10.50)	0.41	0.68	0.18
Comments from others (Oth) (0–16)	15.00 (12.50–16)	15.00 (12–16)	0.45	0.66	0.20
Perceived cognitive abilities (PCA) (0–28)	11.00 (10.50–20.50)	15.00 (8.50–22.5)	0.74	0.46	0.33
Total score (0–148)	75.00 (65–106)	93.00 (59.50–125)	1.21	0.23	**0.54**
**FACT-GP (quality of life)**					
Physical well-being (PWB) (0–24)	16 (10.50–20)	21 (12.50–21.50)	1.89	**0.06**	**0.85**
Social/family well-being (SWB) (0–20)	14.00 (10–17.75)	12 (10–16.63)	−0.73	0.47	−0.33
Emotional well-being (EMB) (0–16)	9.00 (5.50–11.50)	11 (7–12.50)	0.68	0.50	0.30
Functional well-being (FWB) (0–24)	13.00 (6.50–19)	14 (9.50–18.50)	1.13	0.26	**0.51**
Total score (0–84)	49.00 (37.25–65)	57.25 (44.50–64)	0.67	0.50	0.30
**UCLA loneliness scale (20–80)**	30.00 (21.50–52)	31.00 (23–48)	0.68	0.50	0.30

*Notes:* An effect size (r) was calculated from the z value of the Wilcoxon signed-rank test (r = z/√n) and can be interpreted as a small (r ≤ 0.10), medium (r = 0.30), and large (r ≥ 0.50) effect size (Cohen, 1988). *p* < o.o5; near statistical significance 0.05 < *p* < 0.1

#### Acceptability

Descriptive analysis of the satisfaction questionnaire results revealed that most participants (80%) expressed high to very high enjoyment from the program and satisfaction with the CO-OP component addressing everyday problems, with the group intervention, with the psychoeducational content, and with the group setting (via Zoom). Eighty percent of them preferred the program rather than face-to-face treatment, and they would recommend this therapeutic format to another person with the same condition. Despite this, when asked if they would have preferred to participate in individual treatment or group intervention, three participants (60%) answered “individual treatment.” In addition, only three participants (60%) expressed high to very high satisfaction with the intervention in general and with the therapeutic relationship, and only two participants (40%) expressed a desire to continue the treatment if possible.

Qualitative analysis of the responses to the feedback interviews revealed three main themes:(a) group dynamic and therapeutic context, (b) intervention components, and (c) client factors. The themes, sub-themes, and sample quotes are summarized in Supplementary Table S3.

- Group dynamic and therapeutic relationship. The main factors which impacted group dynamic were heterogeneity, inconsistency in participants’ meeting attendance, one participant’s dropout, and preliminary acquaintance with other participants. While some participants found individual differences and the group’s atmosphere to be enriching, others expressed heterogeneity as divisive. The opinions about group discussions were also diverse. While some participants enjoyed sharing in the intimacy of a small group, others commented that the discussions were tedious and dull. Additionally, participants emphasized that the group provided them with opportunities to normalize their experience after cancer by relating to others’ similar experiences and feelings, thus reducing their sense of being “alone.” Finally, participants described the therapeutic relationship positively and mentioned that it contributed to the group’s atmosphere. Some participants mentioned that adding individual sessions would benefit them.
- Intervention components. Participants favorably noted the various components of the intervention. They mentioned that the psychoeducational content helped them to understand the connection between their symptoms after recovering from cancer and cognitive decline. Most participants were highly satisfied with the use of GPDC strategy to achieve their goals. However, some participants said they needed more group sessions to practice the strategy and implement it in their daily life. Furthermore, participants suggested to shorten the duration of sessions to 1 h and to divide the intervention components into two sessions. 
- Client factors. Several personal factors influenced participants’ perceptions of their functional cognitive improvement and engagement with the process. Participants described motivational factors such as wishing to improve their cognitive condition, their belief that the intervention could help them, and their willingness to learn from other participants’ experiences and successes in the group sessions. Also, participants reported that increasing feeling of self-compassion and self-acceptance were supporting factors in increasing their perceived cognitive self-efficacy.

### Potential effectiveness

#### Primary outcome—activity performance in participant’s chosen goals

**Fig. 2:**
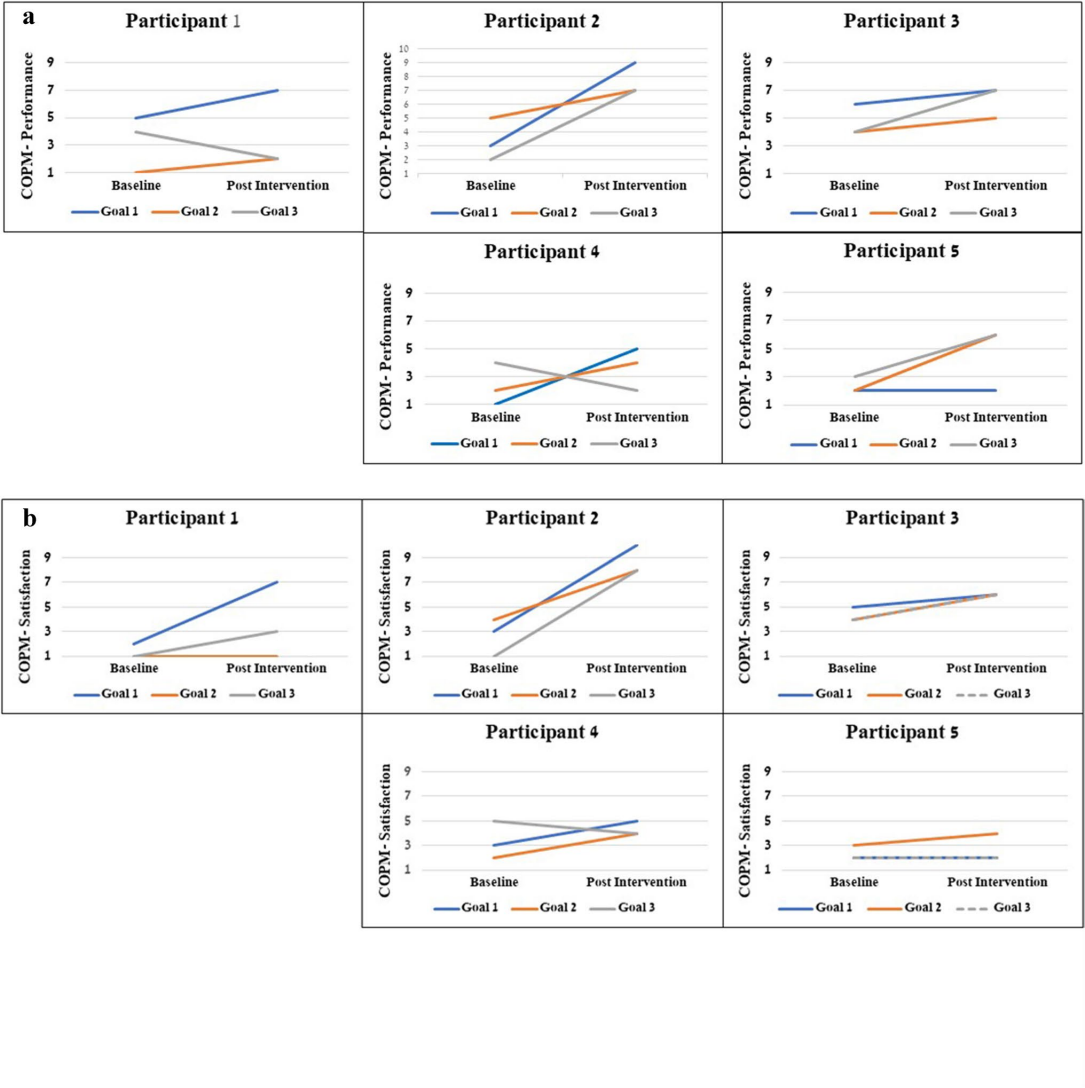
**a** COPM performance scale ratings at baseline and post intervention. **b** COPM satisfaction and satisfaction scale ratings at baseline and post intervention. Note: The goals appear according to the order of their appearance in Supplementary Table S4 (Goal 1 is trained goal)

Each client set three goals at baseline, using the COPM. One goal was directly addressed during intervention (the trained goal); the other two goals were not, thus enabling the assessment of generalization and transfer of learning. In total, 15 occupational goals were set, representing broad participation domains and body functions (see Supplementary Table S4). Overall, each participant reported clinically significant performance improvement in at least one of the three identified goals (see Fig. 2a), and via the COPM, they reported satisfaction with their performance (see Fig. 2b). Overall, 3/5 (60%) trained goals and 8/10 (80%) untrained goals showed clinically significant improvement.

Wilcoxon signed-rank test results (see Table 2) indicated statistically significant improvements from baseline to post intervention, with a large effect size, in COPM performance and satisfaction scale ratings (*z* = 2.02, *p* = 0.04, r(ES) = 0.90), and in PQRS scale ratings (*z* = 2.03, *p* = 0.04, r(ES) = −0.91).

#### Secondary outcome measures—objective and subjective cognitive function, quality of life, and loneliness

The Wilcoxon signed-rank test did not reveal statistically significant differences when examining the changes in all the secondary outcome measures, even though some large effect sizes were found (highlighted in bold in Table 2). One exception was a trend toward significant improvement, with large effect size, was found in the Physical Well-being subscale of the FACT-GP (*z* = 1.89, *p* = 0.06, r(ES) = 0.85). The FACT-Cog total score and PCI subscale score demonstrated a significant clinical improvement for three participants (60%), no change for Participant 2, and a significant clinical decrease for Participant 5 in total score and the PCI subscale score.

## Discussion

The purpose of this study was to examine the feasibility of CRAFT-G in terms of recruitment, retention, adherence, and acceptability and to assess its potential effectiveness in preparation for a large-scale trial. Our results showed encouraging results that demonstrate the feasibility of CRAFT-G intervention according to predetermined outcomes. Potential effectiveness was supported by statistical and clinically significant improvements in occupational performance and reported satisfaction with performance. Likewise, clinically significant improvement was found for subjective cognitive functions.

### Feasibility

#### Recruitment, retention, and adherence

The study’s recruitment and retention rates met the feasibility criteria. This can be compared to a previous study that described the CRAFT results in an individual format [26]. Partial adherence was found to the CO-OP group sessions and the CCT. Four participants (80%) met the adherence threshold of 80% session attendance, a rate lower than those reported in remote group intervention among adults with Attention Deficit Hyperactivity Disorder (ADHD) [73] and in a CO-OP in-person group for adult out-patient rehabilitation [74]. However, our low group rate can be attributed to participant 5’s low attendance, due to personal circumstances. Additionally, two participants (40%) exceeded the minimum 8 h of CCT sessions, in line with a previous study involving home-based CCT, which reported only 25%–30% of participants adhering to at least 66% of CCT-related sessions [75].

#### Acceptability

Overall, quantitative measures indicated a moderate to high level of satisfaction with CRAFT-G, in line with a previous study suggesting that the combined CCT and CO-OP approach is suitable for treating adults with CRCI [26, 34]. Also, remote delivery did not negatively impact general satisfaction, aligning with previous Tele-CO-OP studies [26, 32, 76].

Qualitative findings from the feedback interviews provided deeper insight into individual experiences and pointed to three main themes:

- *Group dynamic and therapeutic context.* The development of cohesion may have been hindered by the relatively short duration of the intervention, participant dropout, and the virtual format [77, 78].
- *Intervention components.* Specifically, participants mentioned that learning about CRCI enhanced their feelings of self-compassion and self-acceptance, which could positively influence their cognitive self-efficacy [34].
- *Client factors* that influenced acceptability were also described in therapeutic group interactions, including participants’ personal factors such as motivation and self-acceptance [77, 79]; along with medical and demographic characteristics such as recovery duration, age, and cognitive status [77].

### Potential effectiveness

Our primary outcomes showed statistically and clinically significant improvements in occupational performance and satisfaction with occupational performance, as reported both by participants and therapist. These results, which are in line with previous adult Tele-CO-OP studies [32, 34, 44], may indicate the effectiveness of CRAFT-G for achieving significant gains in occupational goals.

As for the secondary outcome measures, no significant change was recorded in objective cognitive outcomes when measured by ACE assessment. Considering that the current sample’s cognitive status did not determine a threshold for excluding participants without objective cognitive impairment, (MOCA < 26; [47]), the potential for further improvement by training was relatively weak, due to the ceiling effect [80]. Another explanation could be that the dose of CCT was not sufficient to create an objective effect in cognitive function, given that only two participants completed 8 h of CCT, the suggested minimum to be efficacious [76, 81]. Finally, challenges in detecting subtle cognitive changes through traditional neuropsychological tests assessing CRCI may have accounted for no change in the objective cognitive outcome measure [5].

When examining subjective cognitive function, as reported by the participants using the FACT-Cog, the most cited assessment for reporting the subjective cognitive status among survivors with CRCI [82], our results revealed significant clinical improvement for three participants, no change for one participant and significant clinical reduction for the last participant. A closer look at these results revealed that they correspond to the CCT adherence of each participant: higher FACT-Cog scores were associated with longer training on the CCT. These findings align with previous findings showing that CCT enhances perceived cognitive functions [14, 21, 22].

A trend of significant improvement was found in the physical QoL outcome. This finding is in line with other studies of interventions for CRCI that reported partial effects on QoL, namely, improvement in various domains of QoL while other domains did not improve [34, 37, 42].

The current study aims to contribute to the emerging understanding of the effect of telehealth group interventions on loneliness among adults with CRCI. Our results, which did not show significant improvement in the loneliness measures, are contradictory to another group intervention where a significant decrease in loneliness was reported. The intervention was delivered across three locations, with a live session conducted at one site and broadcast via telehealth to the other two; however, the study did not report comparative data between the two delivery modes [63]. Nevertheless, our qualitative findings are in line with previous data showing that the group dynamic contributes to the normalization of post-cancer experiences, which reduces the “alone” feeling [35, 42]. Our quantitative findings may be attributed to insufficient group cohesion, given the established correlation between cohesion and decreased feelings of loneliness [83, 84].

### Study limitations

Several limitations should be acknowledged in the interpretation of this study’s findings. Firstly, the small sample, which was recruited from one geographical area, limits the generalizability of the study results. Secondly, the eligibility criteria excluding individuals with a psychiatric background posed challenges in recruitment, since this is very common among cancer survivors [85]. In addition, the ACE may not be sensitive enough to detect changes achieved by short-term intervention [5]. Indeed, the data about ACE are scant [53, 67], and it has not been used before as an outcome measure. Lastly, preliminary acquaintance among some of the participants, as well as the diverse sample, might have influenced group dynamics and cohesion. The current study included only six group sessions, as suggested by previous group intervention [42, 63]. Thus, due to our results reporting no change in loneliness and the relatively low cohesion, we recommend future studies to consider expanding the intervention with additional group sessions. These limitations underscore the need for caution in generalizing the study findings, and they highlight areas for improvement in future research designs.

## Conclusion

The current study demonstrated the feasibility and the potential effectiveness of CRAFT-G intervention, indicating that CRAFT-G may be considered an accessible and pragmatic method of remote delivery of intervention to adults with CRCI. Due to the small sample size, the potential effectiveness should be interpreted with caution. With that said, encouraging results regarding potential effectiveness were found in occupational performance and self-perceived cognitive function. However, objective cognitive function, QoL, and loneliness measures did not improve as expected. Future studies on a larger scale are warranted to further evaluate the effectiveness of CRAFT-G.

### Implications for occupational therapy practice

This study suggests that clinicians should consider the following when treating adults with CRCI:

- An occupation-based approach combined with specific cognitive-domain skills training might yield improvements in occupational performance and self-perceived cognitive function.
- Educating CRCI patients about their condition increases satisfaction with intervention, and may improve various aspects of self-perception.
- A remote group delivery format is a feasible approach when treating adults with CRCI.

### What this study added

Findings from this study indicate that remote intervention which includes a combination of CCT and functional group treatment seems to be a feasible and potentially effective option for adults with CRCI, with an aim to improve occupational performance and self-perceived cognitive function.

## Supplementary Information

Below is the link to the electronic supplementary material.

ESM 1 (303 KB PDF)

## Data Availability

The datasets generated during and/or analyzed during the current study are available from the corresponding author on reasonable request.
