# Supplementary material for: Remote group intervention for adults with cancer-related cognitive impairment: a feasibility study
Source: Support Care Cancer. 2025 Dec 4;34(1):5. doi: 10.1007/s00520-025-10114-7 (PMC12678603; doi:10.1007/s00520-025-10114-7)
Supplement: Supplementary file 1 — (303 KB PDF) [file 520_2025_10114_MOESM1_ESM.pdf]

Supplementary Materials

Remote group intervention for adults with cancer-related cognitive impairment: A feasibility study. Supportive Care in Cancer

Authors' names: Chenanit Hamami, Tamar Peretz, Mor Nahum, Talia Maeir, Ofra Maimon and Yafit Gilboa.
Chenanit.hamami@mail.huji.ac.il
1 School of Occupational Therapy, Faculty of Medicine, Hebrew University of Jerusalem, 91240 Jerusalem, Israel.
2 Department of Physical Medicine and Rehabilitation, Hadassah Medical Center, POB 24035, Jerusalem 91240, Israel.

Table S1.
Summary description of the Adaptive Cognitive Evaluation (ACE) subtests

Task	Description	Cognitive Domain	Outcome variable
Triangle Trace (TNT)	This includes two separated tasks, first performed independently and then concurrently. In the Tap task, participants are instructed to attend to a frame of colored shapes and tap when the green triangles (target) appear and ignore all other colored shapes (distracting). In the Trace task, participants are instructed to trace a line with one hand and tap with the other when the green triangle border appears.	Dual task	Mean reaction time (RT)
Stroop/ Color Tricker	Participants view colored words that spell a color and are instructed to identify the color of the word (target) and ignore the word spelled (distractor).	Response inhibition	Overall Rate Correct Score
Task Switch/ Sun & Moon	Participants view a cue (i.e., Color or Shape) followed by a stimulus (i.e., a 'orange-colored' or 'green-colored' sun, or moon), and are instructed to identify the feature type cued. For instance, if a participant is cued Color, and is shown a green sun, participants indicate green by pressing the green button on the screen.	Task switching	Overall Rate Correct Score
Flanker	Participants view an array of five arrows and are instructed to identify the central arrow (target) and ignore the flanking arrows (distractors). The four flanking arrows are always the same as each other. The central arrow can match them or face the opposite direction.	Selective attention and interference resolution	Overall Rate Correct Score

The information was retrieved from https://neuroscape.ucsf.edu/researchers-ace/

Figure S1. *The study procedure*.

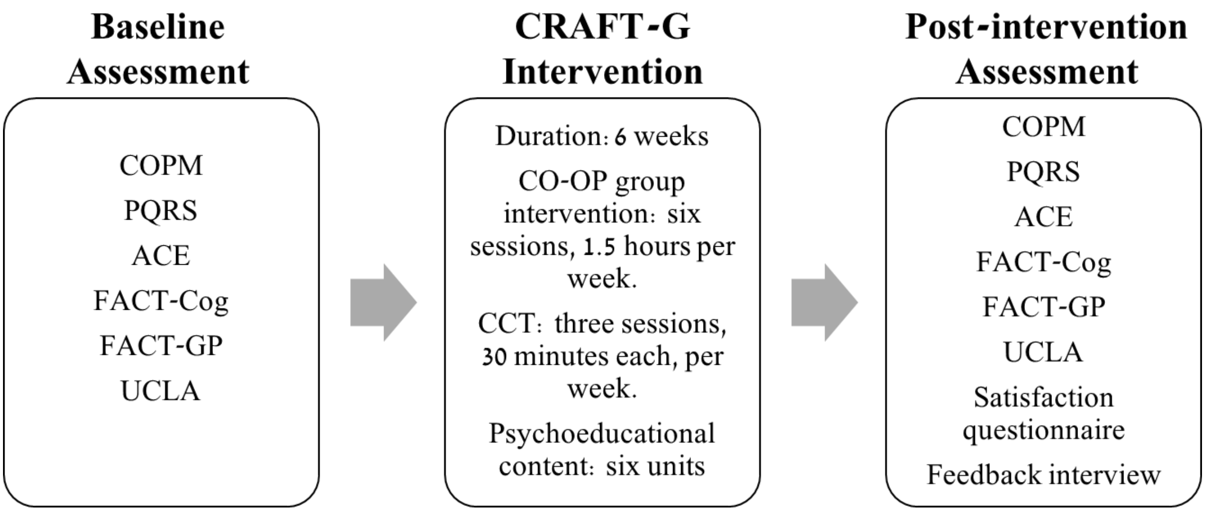

Notes. COPM: Canadian Occupational Performance Measure; PQRS: The Performance Quality Rating Scale; ACE: Adaptive Cognitive Evaluation; FACT-Cog: Functional Assessment of Cancer Therapy–Cognition; FACT-GP: Functional Assessment of Cancer Therapy–General Practice; UCLA: The revised UCLA Loneliness Scale; CRAFT-G: Computerized Retraining and Functional Treatment Group.

Table S2.

CRAFT-G protocol: Psychoeducational content, group stages, and CO-OP goals according to the sessions

Session	Psychoeducational Content	Stage of group formation	CO-OP goal pursuit
1	CRCI: prevalence, causes, diagnosis, consequences<br><br>Introduction to the principles of CRAFT-G intervention	Forming:<br>• Meeting other group members<br>• Defining group purpose and rules<br>• Identifying individual strength and weaknesses	Learning about Goal-Plan-Do-Check
2	Cognitive functions, executive functions	Storming:<br>• Competition among group members<br>• Engaging in conflict from desire to succeed and avoid failure in front of new friends or strangers	Guided discovery of individual DPA-specific strategies
3	Neuroplasticity and CCT:<br>Refine the principles of treatment and its effect on improving cognitive function		
4	Side-effects after treatment, and their connection to cognitive decline	Norming:<br>Developing cohesion with greater sharing, turn-taking, and emergent collaborations on problem- solving	Individual goals shifting toward group support
5	Mental conditions (depression, stress, anxiety) and psychosocial aspects (self-stigma and loneliness) after recovering from cancer, and how it is relevant to CRCI		Teamwork and group goals
6	Review and summary of psychoeducational content	Performing:<br>• Engaging in collaborative projects<br>• Confidently defining plans and testing themes in public	Generalization and transfer of strategies to preserve successes in daily life

Note. The four stages of group formation are adapted from Tuckman (1965). CO-OP = Cognitive Orientation to Occupational Performance. DPA = dynamic performance analysis.

Table S3.
Themes, sub-themes and quotes emerging from participants' feedback interviews.

Theme	**Group dynamic and therapeutic relationship** Sub-themes	Sample Quotes
	About the therapeutic relationship	Participant 1*:" I think that she [the facilitator] contributed a lot, she has a lot of compassion and is very practical, and she both empathizes and shares how she does or does not succeed in her goals, so there was a pleasant atmosphere in the group."* Participant 5: *"We're a bit like children sometimes; there are symptoms, there are side-effects... Maybe it is necessary to put the finger more on the pulse. That is, to pay attention that everyone is connected."*
	About the group's composition	Participant 4:*"There was one person there that I really can't stand. She's not a bad person, but I just can't stand her. In any case, it kind of... made the group dynamic difficult for me."* Participant 3: *"I think that maybe for the young girl who was there, it would have been good to have older women who would have greatly encouraged her, not me. As for me, I don't think there's an advantage to having someone young there."*
	About the group's atmosphere	Participant 1: *"Participant 3 said that the group was a bit depressing, because she heard from someone relatively young talking about how having cancer has affected her life. I'm glad that I can empower Participant 2; I'm not depressed hearing about the difficult things she has been through. I do feel a sense of compassion towards her. It's a bit unfortunate that people who started then left immediately, but it's part of the dynamics of a group and something new, so it was a bit disappointing. The downside is that people start and then don't continue, and they're not committed to it, which is upsetting. On the other hand, there's intimacy in a small group."* Participant 5: *"I saw that I have something to give and something to take from the group... It's a reciprocal relationship, meaning both taking and giving... Even when I was in a conversation, I had something to contribute as well... I felt that there is something to gain in the research, and it helped me."*
	Relating to similar feelings and experiences	Participant 2: *"I think that the ability of the group is to support each other, to uplift one another. Doing it alone won't always yield the same results."* Participant 5: *"When I say 'the power of a group,' it means that when you hear someone talking about their difficulties, you already experience some kind of identification. It means you already think 'okay, I'm not alone in this story; there, she's going through what I'm going through.' ...I'm not imagining, I'm not going through something here where no one can understand me. So, in itself, this is a very powerful thing."*
Theme	**Intervention components** Sub-themes	Sample Quotes
	CCT	Participant 2*: "I love games, so for me, it was a competitive thing. It was always about trying to do better than what I did the first time, so I really enjoyed it."* Participant 1: *"I did feel there is something about it to do with neuroplasticity. Like something awakened that was dormant."*
	CO-OP component	Participant 2: *"I also learned through the program how to execute the plan [from GPDC strategy] – setting a goal, checking the goal – I think yes, I took a lot of things from there - I use them in my daily life as tools."*

		Participant 3: *"I think it was very good. We learned something, we were sent to implement it either during the activity or throughout the week. There was an invitation to participate in it, to follow it, meaning to take it very, very seriously. Yes, so I think it was very focused on the implementation and success of new things."*<br><br>Participant 4: *"I found it important that the therapist emphasizes the goal; that it needs to be quantified, as if it can be measured. And I remained with a feeling like... how do you implement this, yes...? How do you implement this idea, and especially how do you implement the check [from GPDC strategy] phase?"*
	Psychoeducational content	Participant 1: *"[The content] gave a 'gushpanka' [seal of approval] to the theory, showing that you really didn't make it up – and I'm not alone in that matter."*<br>*Participant 2: "The presentation wasn't long, and we could have finished it – but because we lingered a lot on ourselves, and also on how we're implementing the strategy, when we got to the presentation, we kind of had to rush it."*
Theme	**Client factors**<br>Sub-themes	Sample Quotes
	Motivation for intervention	Participant 1: *"I didn't make it up; there are studies, it's a thing, it's a real field, people are working on it. So, all of this is a kind of drive and hope and motivation to grasp yourself and change."*<br><br>Participant 4: *"I managed to maintain a day-on, day-off schedule for cognitive training, which I didn't achieve this week, by the way. The group ended, and my motivation to play the game significantly decreased."*<br>Participant 5: *"I have a lot of side-effects, and one of them is terrible fatigue. And sometimes it's like I have no energy... I have no energy now to play the game [CCT]; I have no energy now to connect to Zoom."*<br>Participant 1: *"There are basic elements of playing computer games, and I had zero knowledge. Now, Participant 2 gets it; maybe she spent hours playing computer games when she was sick, so she did empower me, as it was fun for her."*
	Subjective perception on cognitive functional abilities	Participant 3: *"Once I was like this, when preparing to leave the house – making lists in my head of what I need to accomplish... 'Don't forget these papers, put on makeup, do this, deodorant.' And then in the group, I said to myself, 'What nonsense; don't I know that I need to put on deodorant?' ...It's true that sometimes I still need to remember to take certain papers or something like that, but there's something in me that calmed down, and it's not as if automatically doing this checklist and going around with it is something that takes care of me. There are some habits that have been established, and I can rely on myself more."*<br><br>Participant 4: *"Truly I have more appreciation for my coping with the cognitive impairment. I am prouder of myself. I also notice that I pay more attention to the strategies I adopted even before the group... not letting things slip my mind... I am prouder of myself, which releases me from a lot of guilt."*

Table S4.

Participants' selected goals, importance ratings, and classification according to the ICF.

		Goals	IR	Life domain- ICF
P1	Trained	Plan and manage daily schedule	10*	d2301: Managing daily routine
	Untrained	Arrange and plan timetable for writing a book	10	d2101: Undertaking a complex task
	Untrained	Increase working hours' range	10*	b1400: Sustaining attention
P2	Trained	Manage and remember daily schedule	7*	b1642: Time management
	Untrained	Recall location of essential items	10*	b144: Memory functions
	Untrained	Concentrate on one task until completed	9*	d210: Undertaking a single task
P3	Trained	Recall details from a reading book for short-term period	8	b1442: Retrieval of memory
	Untrained	Retrieve details of patients	8*	b1442: Retrieval of memory
	Untrained	Maintain key points of a study article for a long-term period	8*	b144: Memory functions
P4	Trained	Retrieve daily tasks when required	10*	b1442: Retrieval of memory
	Untrained	Be wakeful among people	8*	b110: Consciousness functions
	Untrained	Retrieve details of acquaintances	7	b1442: Retrieval of memory
P5	Trained	Read 15 pages from a book in a day	7	b1400: Sustaining attention
	Untrained	Concentrate on one task until completed	8*	d210: Undertaking a single task
	Untrained	Retrieve details from previous conversations	8*	b1442: Retrieval of memory

Notes. P = Participant; IR = importance rating, rated by participants on a 10-point scale (1:"not at all important," 10:"very important") on the COPM; ICF, the International Classification of Functioning, Disability and Health (WHO, 2001) (http://apps.who.int/classifications/icfbrowser/).
*Indicates goal improved to criterion (≥2 points; Carswell et al., 2004) at post intervention based on participants' COPM performance and/or satisfaction ratings.
